# The Role of Orthobunyavirus Glycoprotein Gc in the Viral Life Cycle: From Viral Entry to Egress

**DOI:** 10.3390/molecules30030503

**Published:** 2025-01-23

**Authors:** Han Gao, Dengshuai Zhao, Canyuan Li, Menghua Deng, Gan Li, Shengfeng Chen, Mengmeng Zhao, Limei Qin, Keshan Zhang

**Affiliations:** School of Animal Science and Technology, Guangdong Provincial Key Laboratory of Animal Molecular Design and Precise Breeding, Foshan University, Foshan 528225, China; gh1@fosu.edu.cn (H.G.);

**Keywords:** orthobunyavirus, Gc protein, viral entry, viral penetration, viral egress

## Abstract

Orthobunyavirus refers to the virus members within the Genus Orthobunyavirus, which is the largest virus genus in the Family Peribunyaviridae and even Class Bunyaviricetes. To date, over 130 species of Orthobunyaviruses have been identified worldwide. Orthobunyaviruses mainly infect arthropods, while some species are capable of being transmitted to mammals, including humans, via intermediate vectors. As emerging and re-emerging pathogens, orthobunyavirus poses a significant threat to both human and veterinary public health worldwide. Currently, there are no commercial vaccines against orthobunyavirus. The structure of orthobunyavirus is relatively simple, consisting of a typical tri-segmented negative-sense RNA genome that encodes four structural proteins (L, Gn, Gc, and N) and two non-structural proteins (NSm and NSs). The highly glycosylated Gc protein, which has a complex conformation and forms polymers embedded in the viral envelope, plays a critical role in inducing neutralizing antibodies throughout the orthobunyavirus infection cycle from entry to egress. This review provides a comprehensive summary of the virus-encoded Gc protein and its role in the virus life cycle from viral entry to egress, offering researchers with valuable integrated information for further investigations.

## 1. OB Disease and Epidemiology

Orthobunyaviruses (OBVs), commonly referred to as viruses belonging to the Genus *Orthobunyavirus*, are members of the Family *Peribunyaviridae* within the Order *Elliovirales* and Class *Bunyaviricetes*, representing the largest among the eight genera within the *Peribunyaviridae* family, with at least 130 species identified to date [1,2]. Humans are susceptible to at least 30 of these viruses [3]. OBV is found globally and has been documented to have a broad spectrum of insect and vertebrate hosts, including humans and ruminants, by hematophagous arthropods such as mosquitoes and midges. Some OBVs, like Tete group OBVs, can be transmitted via ticks [1,4]. These arthropods can reacquire OBV from infected vertebrates, thereby facilitating the insect–vertebrate–insect transmission cycle. Many OBVs pose a threat to human and veterinary public health, reflecting the “One Health” concept.

OBV infections typically present with a range of symptoms, including high/hemorrhagic fever, manifestations affecting the central nervous system (CNS), and reproductive abnormalities (Table 1). Various OBVs, such as Batai virus (BATV, Bunyamwera serogroup), can cause influenza-like symptoms and are disseminated globally by *Anopheles* and *Culex* mosquitoes [5,6,7]. Ngari virus (NRIV, Bunyamwera serogroup), which was responsible for severe outbreaks in Africa in the past century, can lead to high/hemorrhagic fever [8,9,10,11]. Other OBVs, including Fort Sherman virus (FSV), Germiston virus (GERV), and Xingu virus (XINV), have also been reported to infect humans, causing high and/or severe hemorrhagic fever [3,12,13,14]. Furthermore, a significant number of OBV infections are associated with CNS diseases. For instance, Oropouche virus (OROV, Simbu serogroup) is prevalent in Central and South America, transmitted mainly by mosquito bites. OROV presents with high fever and headache, frequently accompanied by aseptic meningitis, resembling dengue virus (DENV) infection [15,16,17,18].

Additionally, viruses within the California encephalitis serogroup, including California encephalitis virus (CEV), Jamestown Canyon virus (JCV), La Crosse virus (LACV), and Ťahyňa virus (TAHV), as well as Snowshoe hare virus (SSHV) and Inkoo virus (INKV), are primarily vectored by *Aedes* and *Culex* mosquitoes and can induce varying degrees of high fever, encephalitis, focal neurological dysfunction, and severe outcomes like seizures and central respiratory failure, particularly in pediatric patients [28,29,30,31,39,43,44]. In addition, numerous OBV infections in pregnant mothers can lead to postnatal CNS abnormalities in the fetus, particularly among ruminant hosts. For instance, the Schmallenberg virus (SBV, Simbu serogroup) outbreak in Europe in late 2011 rapidly spread to thousands of farms, resulting in fever, diarrhea, and abortions with fetal malformations in ruminants, causing significant economic losses [33,34,35,45,46,47,48]. Similarly, Akabane virus (AKAV, Simbu serogroup) can induce high fever, abnormal parturition, and fetal malformations such as brain abnormalities or even anencephaly in ruminants (Arthrogryposis–Hydranencephaly syndrome, A-H syndrome) [22,49,50,51]. In addition, inoculation with genotype I AKAV strains has been linked to cerebral edema and encephalitis in suckling mice and hamsters [21,52]. Analogously, Cache Valley virus (CVV, Bunyamwera serogroup) can also lead to developmental malformations or stillbirths [25,26,53]. These instances highlight the diverse adverse effects of OBV infection, including high fever, CNS symptoms from vector transmission, and maternal infections affecting fetal development, resulting in abortion storms and stillbirths.

In recent years, the continuous identification of novel OBVs has significantly broadened our comprehension of this virus family. This growth is driven by extensive research and advancements in high-throughput diagnostic technologies. The complex interplay among factors like large-scale livestock farming or climate change have also contributed [3,54]. According to the International Committee on Taxonomy of Viruses, the number of classified OBV species has nearly tripled from fewer than 50 in 2011 to over 130 in 2024. The Family *Bunyaviridae* has been elevated to the Class *Bunyaviricetes*, reflecting a deeper understanding of bunyaviruses [1]. Furthermore, nine newly discovered OBVs, including Ntwetwe virus (NTWV) and Yacaaba virus (YACV), remain unclassified [55,56]. The expanding spectrum of OBVs and their associated health risks underscore the need for further research.

## 2. OBV Molecular Properties

**Figure 1:**
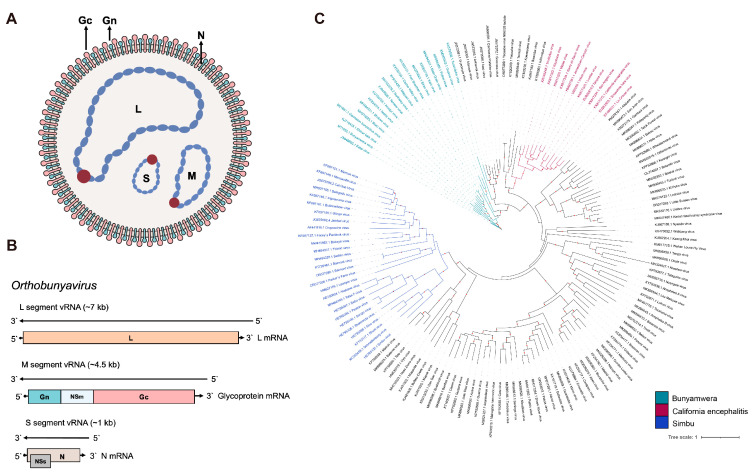
Schematic diagrams of OBV (**A**) viral particle and (**B**) viral genome. (**C**) The global ML phylogeny of all publicly available OBV species based on the Gc region. The GTR + F + I + G4 nucleotide substitution model was used for phylogenetic tree construction. The scale bar represents the number of substitutions per site along the branch in the tree topology. Red dots on the tree topology indicate a bootstrap value over 90. The three biggest serogroups, Simbu, California encephalitis, and Bunyamwera, are labeled in blue, green, and magenta, respectively.

OBV is a tri-segmented, negative-stranded RNA virus whose genome is primed with “cap snatching” from host cellular RNAs. The viral genome is composed of three segments, designated as L (large, ~7 kb length), M (medium, ~4.5 kb length), and S (small, ~1 kb length) according to their sizes (Figure 1). The L segment encodes RNA-dependent RNA polymerase (RdRp), and its upstream region encodes an endonuclease domain crucial for cap-snatching. The M segment encodes a highly glycosylated protein precursor. During the replication and packaging process of the virus, this glycoprotein precursor is cleaved into Gn (N-terminal encoded), Gc (C-terminal encoded), and a non-structural protein, NSm. The S segment encodes N protein, with a frameshift-expressed non-structural protein, NSs, within its open reading frame.

As a tri-segmented RNA virus, OBV can undergo genome reassortment during evolution or complicated co-infections. Furthermore, OBV reassortment can lead to varying virulence among strains, as seen with members of *Orthobunyavirus bunyamweraense* species, including BATV, GERV, NRIV, XINV, and Mboké Virus (MBOV), highlighting the significance of reassortment in shaping the pathogenic potential of OBV [6,11]. While certain studies mentioned that reassortment is confined to viruses within the same species, previous research indicated that reassortment between SBV and OROV based on minigenome-reporter and virus-like particles is feasible, and there may be potential new Simbu viruses emerging under conducive epidemiological settings [57]. This interspecies reassortment potential further enriches the complexity of the OBV etiology.

OBV genomes share similarities and differences with other genera in *Peribunyaviridae* [58]. For example, while OBV encodes NSm and NSs, Pacuvirus and Shangavirus do not encode NSs, and the NSm of the newly discovered Griffinivirus is not described. The remaining four genera, Herbevirus, Khurdivirus, Lakivirus, and Lambavirus, neither encode NSs nor NSm. The Gc encoded by the virus members of these four genera also exhibits a truncated expression characteristic compared to other viruses in this family.

Within the Family *Peribunyaviridae*, all reported zoonotic viruses capable of infecting humans belong to OBV. OBV is usually classified into 18 serogroups according to a neutralization test and a hemagglutination inhibition test [2]. The antibodies to the proteins that inhibit hemagglutination and those that have neutralizing activity were elicited by the viral glycoproteins, especially the Gc of OBV. Since the OBV Gc has an important role, this review introduces the latest advancements regarding its role in the entire infection cycle, including virus–host interactions, covering virus binding, fusion, and egress.

## 3. OBV Gc Protein

OBV virion particles exhibit a spherical or polymorphic morphology, with diameters typically ranging from 80 to 120 nm. The viral envelope surface is covered with a dense array of transmembrane glycoprotein heterodimers composed of Gn and Gc subunits [32,59,60]. These heterodimers are arranged so that every three Gn-Gc pairs form a transmembrane homotrimer, creating a prominent peplomer structure reminiscent of spikes like coronavirus spike trimers. These peplomers play essential roles in mediating the virus–host interaction, facilitating membrane fusion during viral invasion and participating in key biological processes associated with viral replication and pathogenesis. Additionally, Gn-Gc heterodimers, particularly Gc, serve as the primary targets for neutralizing antibody responses, underlining their critical importance in the immune response [61,62,63]. Currently, there is a lack of detailed analysis regarding the surface structure of OBV virus particles. The research findings indicate that in Rift valley fever virus (RVFV), the heterodimer formed by Gn-Gc constitutes the outer shell of the virus [64,65]. Gc-Gc contacts may form a Gn-independent inner shell, which is responsible for virus membrane fusion. This suggests that the outer shell of OBV may have a similar surface Gn and Gc structure to RVFV. However, unlike RVFV, which encodes Gn and Gc of approximately the equivalent size, OBV’s Gn (~32 kDa) size is significantly smaller than Gc (~115 kDa). Further investigation is needed to elucidate how the Gc protein affects the viral surface structure of OBV.

**Figure 2:**
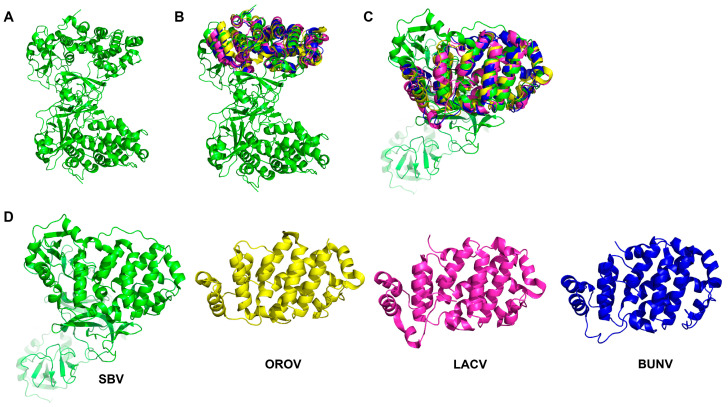
The OBV Gc structure of (**A**) SBV Gc (PDB ID: 6H3S); (**B**,**C**) show the high similarity of OBV head domains from the protein structure alignment. (**D**) shows separate head domain views of SBV (green, PDB ID: 6H3S), OROV (yellow, PDB ID: 6H3X), LACV (magenta, PDB ID: 6H3W), and BUNV (blue, PDB ID: 6H3V).

Although the core region of Gc is generally conserved in OBV, the variable region of Gc exhibits diversity (Figure 2). This variable region of Gc can be further subdivided into a head domain and two stalk subdomains. The head domain forms a spike structure and serves as the primary target for neutralizing antibodies [61,63]. Studies have shown that the glycoprotein Gc belongs to class II of membrane fusion proteins, which is homologous to the fusion glycoproteins of flavivirus and Alphavirus. LACV Gc shares a similar structure with class II fusion glycoproteins of Chikungunya virus, indicating a similar entry mechanism related to cellular cholesterol levels between these two viruses. In addition, mutants on the conserved histidine and alanine of the *ij* loop in LACV Gc can downregulate viral entry and replication, revealing the important role of OBV Gc. Similar findings were also revealed for SBV and even RVFV [63,66,67,68,69,70]. Interestingly, a mutant virus with N-terminal partial deletion of BUNV Gc can still replicate in BHK-21 cell culture, indicating that the core region of OBV Gc itself has the necessary membrane fusion function rather than the variable region [71]. Of course, changes in Gc variable regions can affect the efficiency of virus infection, but variable regions are not necessary for OBV infection. This is also consistent with the phenomenon of the Gc-Gc inner shell responsible for membrane fusion in RVFV research, indicating that OBV may also have a very similar viral particle structure. The fusion peptide, on the other hand, is located within the conserved C-terminal portion of Gc [63,66,67,68,69]. In addition, there is a transmembrane region and cytoplasmic tail at the C-terminus of BUNV Gc, whose changes can affect the assembly and structural morphology of viral particles, and they also have important biological functions [61,72].

## 4. Gc-Mediated Viral Entry

Like other viruses, OBV must first enter host cells to initiate infection. The viral invasion is usually primed by the interaction between the viral surface proteins and host receptors. These receptors are usually located on cell membranes, which may be glycoproteins, lipoproteins, and even proteases like MERS-CoV receptor DPP4 [73]. Moreover, from an evolutionary perspective, a virus does not rely solely on one receptor molecule to mediate virus invasion. This exemplifies the multi-pathogenic nature of viral entry. For example, COVID-19 has been reported to use the main receptor ACE2 and the auxiliary receptor TMPRSS2 to mediate virus invasion. In addition, NRP-1, AXL, and HDL scavenger receptor type 1 have also been reported as SARS-CoV-2 receptors [74,75,76]. OBV mediates membrane fusion and invasion through glycoproteins, especially Gc. However, there are relatively few reports on the corresponding receptors and receptor-related factors. Unlike specific receptors such as RVFV (Lrp1) and severe fever with thrombocytopenia syndrome virus (SFTSV and CCR2) that have already been identified, there is very little information about bona fide receptors for OBV [77,78,79]. Some studies suggest that Lrp1 not only acts as a receptor for RVFV but also as a key cell surface factor mediating OROV invasion, indicating the widespread importance of Lrp1 in various bunyavirus infections [77,80].

In addition, certain broad-spectrum molecules that are widely reported in various virus invasions have been reported to play a role in OBV invasion. Some cell surface glycoproteins can prime the invasion of OBV. Heparan sulfate (HS) is an amino polysaccharide widely expressed on the cell surface, extracellular matrix, and basement membrane. HS has been reported to be involved in the invasion of various viruses. For example, the viral entry of hepatitis B virus (HBV) also involves the low-affinity binding of HS proteoglycans (HSPGs). HBV first binds to HSPGs on the surface of liver cells, and this low-affinity interaction can stabilize the virus’s position and promote high-affinity binding between the virus and receptors such as NTCP. This binding mechanism helps HBV effectively enter and infect hepatocytes [81,82]. HS also assists in infections by viruses such as SARS-CoV-2, herpesviruses, porcine reproductive and respiratory syndrome virus, and DENV [83,84,85]. In the context of OBV, HS has been linked to the infection of Simbu serogroups SBV and AKAV [86,87]. Heparinase-treated cells or HS knockout cells are employed for the infection of SBV or AKAV, and Gc-mediated OBV viral entry is impaired. The VSV pseudo-typed virus bearing AKAV glycoproteins (Gn and mutant Gc) was also used for validation in related studies, and the site mutant on Gc receptor-binding regions helped identify key residues on the AKAV Gc that are responsible for HS affinity. This indicates that the HSPG-mediated cell adhesion of OBV is crucial for viral invasion.

Similarly, C-type lectins, such as DC-SIGN, are also a class of co-receptors that bind to viruses and have been reported in viral invasion, including Japanese encephalitis virus, human immunodeficiency virus, and hepatitis C virus [88,89,90]. Some OBVs have also been reported to use DC-SIGN to infect cells, especially dendritic cells and macrophages located on the skin, which anatomically co-occur with transmission through arthropod bites. For example, LACV, GERV, and even SFTSV have been reported to facilitate viral infection using DC-SIGN [91,92]. Other C-type lectins, including Mincle, Dectin-1, and Dectin-2, may also act as co-receptors when OBV invades cells. HSPG is considered an attachment receptor, but whether lectins are attachment receptors or entry receptors requires further identification [93]. Furthermore, the highly glycosylated nature of OBV Gc facilitates its capture by DC-SIGN and other molecules, thereby aiding OBV viral entry. However, cell lines lacking C-type lectins are still susceptible to OBV, indicating that OBV also has alternative mechanisms for cell invasion.

## 5. Gc-Mediated Membrane Fusion and Viral Penetration

After interacting with cell surface receptors, OBV initiates intracellular uptake to complete the invasion of viral particles. Although detailed analyses of OBV Gc function remain limited, studies indicate that cholesterol depletion using methyl-β-cyclodextrin disrupts the invasion and infection of AKAV and OROV [94,95]. This suggests that cholesterol and other lipids are essential for the formation of receptor-rich microdomains, facilitating OBV uptake, and the formation of early endosomes, which is also consistent with the fact that Gc is classified as class II fusion proteins [61,63,70]. In addition, studies have shown that the intracellular motifs of cellular receptor proteins play a crucial role in the absorption of viral particles in vivo, and DC-SIGN encodes such motifs. The OBV Gc can anchor these motifs and play a role in the process of virus endocytosis [96]. Studies have shown that clathrin-mediated endocytosis also plays a role in the invasion of several OBVs into cells. LACV has been validated to enter Hela cells and primary neuronal cells through dynamin- and clathrin-dependent endocytosis. Confocal assays revealed that the LACV Gc protein is highly colocalized with clathrin [97]. Similarly, findings have been reported for OROV entry into Hela cells, which also involves clathrin-mediated endocytosis [95]. Moreover, AKAV has also utilized the dynamin- and clathrin-dependent endocytosis pathway for cell entry, as evidenced by experiments involving clathrin inhibitors [94]. This highlights the complex mechanism underpinning OBV invasion, where auxiliary pathways may still influence the ability of OBV to enter cells and establish infections. Overall, further research is essential to comprehensively describe the infection process of OBV by analyzing the specific cell surface receptors targeted by OBV Gc. While OBVs share numerous similarities, substantial differences may still exist in the invasion strategies employed by certain viruses and their corresponding target cells.

## 6. Gc Pre-Maturation Under Endosome Acidification Facilitates Entry

**Figure 3:**
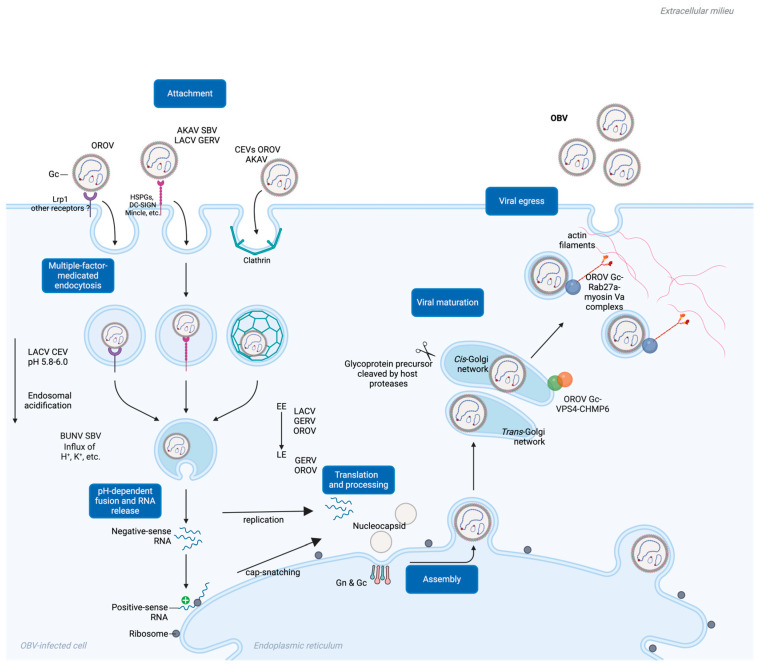
A schematic diagram of a comprehensive mechanism view of Gc’s roles during OBV viral entry. EE, early endosome; LE, late endosome.

The fusion of the viral envelope with the cell membrane is the final step for enveloped viruses to enter the host cell, allowing for the release of the viral genome into the cytoplasm (Figure 3). The conformational changes in Gc required for membrane fusion are partially dependent on the trigger by endosomal acidification. Similarly to viruses such as influenza A virus and rotavirus A, pH-dependent endosome acidification also plays an important role in OBV infection [98,99]. A pH value in the range of 5.8–6.0 was determined for beneficial LACV and CEV entry [100,101]. OBV virus particles enter cells through the endocytosis mechanism, reaching endosomal vesicles, where they subsequently fuse with the cytoplasm through membrane fusion. The transportation of viruses from early endosomes to late endosomes is complex and dynamic, with the endosomal pH value decreasing from about 6.5 in early endosomes to approximately 5.5–5.0 in late endosomes. Research indicates that the infection of certain OBVs, such as AKAV and OROV, relies on endosomal acidification and is sensitive to pH-altering drugs, including chloroquine, NH_4_Cl, and bafilomycin A1 [95,97].

Studies have shown that Rab5 is crucial for early endosome transport and maturation. Its DN mutant blocks LACV infection, indicating that LACV infection relies on early endosome transportation. Similarly, GERV and OROV have been validated through confocal assays to be transported through early endosomes. However, in terms of late endosomes, different OBVs exhibit different characteristics. For example, the role of Rab7, a key enzyme in late endosome maturation, varies among different OBVs. For example, LACV infection does not appear to rely on active Rab7, while OROV can enter Rab7-modified endosomes [95,97]. GERV is also reported to enter cells via late endosomes [102]. This suggests that certain viruses may utilize various mechanisms to escape endocytosis before reaching the late endosome stage, such as during the sorting of early endosomes into multivesicular bodies. Similar strategies may be employed by other OBVs, including Crimean–Congo hemorrhagic fever virus (CCHFV) [103].

In addition to endosomal acidification, other ions’ levels also play important roles in OBV infection. Class II fusion proteins act as indicators of endosomal acidification via their conserved histidine, frequently determining the optimal pH level for viral fusion. For example, the maturation from early to late endosomes is accompanied not only by changes in H^+^ levels but also by changes in other ion concentrations, such as K^+^ [104,105]. Recent studies have shown that SBV and BUNV both rely on endosomal K^+^ influx for successful viral infection. Treatments that inhibit K^+^ influx can impair SBV and BUNV infection. However, the precise roles of K^+^ and Na^+^ in OBV infection still require further investigation. Notably, the role of K^+^ may differ among various Bunyaviruses. For example, K^+^ accumulation is crucial for BUNV infection and is regulated by cellular cholesterol abundance, whereas K^+^ ion carriers have been reported to inhibit LACV infection [106]. These discrepancies suggest that OBV may adopt different strategies to utilize their endosomal environment to achieve infection and replication.

## 7. Gc Maturation in the Golgi Apparatus and Its Role in OBV Egress

The invasion mechanism of OBV involves multiple complex mechanisms, among which the glycoproteins encoded by the M gene, especially Gc, play a key role. While the translation of L and N proteins are in cytoplasma-free ribosomes, Gc and Gn translation require ER-bound ribosomes [58]. The maturation process of the OBV Gc in the cytoplasm, particularly during the transition from the endoplasmic reticulum (ER) to the Golgi apparatus, remains unclear. However, other non-OBV bunyaviruses may provide more information [107]. For example, after the glycoprotein precursor of CCHFV is produced from the ER, the precursor is cleaved into mucin-GP38-Gn-NSm-Gc at the RRLL-RKLL-RKPL residues in the *cis*-Golgi network. The CCHFV Gc matures in the *trans*-Golgi network and subsequently participates in the assembly of mature CCHFV particles [108]. In OBV, the M gene of LACV can be cleaved by serine proteases (SPase) in host cells, facilitating the formation of mature Gn and Gc, thereby assisting in the completion of LACV invasion. The BUNV Gc is also cleaved by host SPase and signal peptidase in the Golgi apparatus, with each cleavage site located before the 5′ end of each mature fragment cleaved by Gn/NSm/Gc. After cleavage, Gn and Gc still need to undergo modification in the Golgi apparatus to reach maturity [107,109]. Related studies have also mentioned that the Gc of SBV and OROV has undergone a similar maturation mechanism, benefiting OBV invasion. Although detailed information regarding the precise cleavage mechanism is not yet available, similar mechanisms may be shared among various OBVs.

During the OROV Gc’s maturation and viral assembly process, the intracellular endosomal sorting complexes required for transport (ESCRT) machinery are recruited onto the Golgi apparatus [110]. The ESCRT complex components located on the Golgi apparatus, such as VPS4, are essential for OBV maturation, budding, and virus particle release. In particular, confocal assays and co-immunoprecipitation assays have demonstrated that OROV-encoded Gn and Gc interact with the ESCRT-III component CHMP6. The overexpression of the dominant-negative form of CHMP6 significantly reduces OROV Gc secretion, indicating that CHMP6 plays a crucial role as a key factor in the germination process of OROV. Gc also plays an important role in the budding and release of OBV from infected cells. OROV-encoded Gc can achieve packaging maturation and budding through Rab27a GTPase and its effector myosin Va [111]. The envelope glycoprotein complex of OROV is synthesized in the ER and subsequently transported to the Golgi apparatus following virus assembly. During this process, Rab27a contributes to the formation of OBV-induced structures and the transport of viral particles by interacting with OROV glycoproteins. The involvement of effector proteins, such as Myosin Va, further promotes the transport of virus particles along actin filaments toward the cell membrane, ultimately facilitating the release of mature OROV particles through exo-membrane fusion to the cell surface.

## 8. Conclusions and Perspectives

Typically, OBV is a class of viruses primarily hosted by insects that also exhibit zoonotic potential, demanding further exploration into the mechanisms underlying their cross-species transmission via insect bites to mammals and humans. Additionally, understanding how OBV modulates the immune systems of arthropod hosts could yield valuable insights, similar to research on flaviviruses like DENV and Zika virus [112]. As our understanding of OBV advances, new members of the *Bunyavirales* class are being rapidly identified, demonstrating the dynamic and evolving nature of virological research [1].

Prominent members of the OBV family, such as OROV and LACV, have significantly impacted humans, especially in the Americas, while SBV and AKAV can cause considerable losses in ruminants and related agricultural industries. However, current research is primarily focused on epidemiological aspects, with limited insights into the mechanisms underlying OBV, particularly the crucial role of the glycoprotein Gc in the viral cycle. Extensive prior research on bunyaviruses from other viral families like RVFV and SFTSV could enhance our understanding of OBV, while a deeper exploration of OBV will broaden our perspective of bunyaviruses as a whole.

The importance of studying OBV-encoded Gc lies in its role as a key target for inducing neutralizing antibodies and viral membrane fusion, guiding the development of diagnostic methods, serological epidemiology, and vaccines [113,114,115,116]. Animal experiments have shown that the amount of OBV-neutralizing antibodies in serum antibodies induced by Gc head domain mutants is significantly reduced compared to wild-type Gc head domain mutants, indicating the importance of the head domain in inducing neutralizing antibodies of SBV. N-glycosylation sites and disulfide bonds located in the Gc head domain are also considered crucial in eliciting neutralizing antibodies [117,118]. In addition, studies have identified that the two N-terminal domains of AKAV Gc have the ability to induce high-level neutralizing antibodies in mice, indicating the important role of Gc research in vaccine development, especially the feasibility of subunit vaccines that use the OBV Gc variable region as the main antigen. In addition, studies have reported the use of viral vectors such as EHV-1 to construct recombinant vaccines based on Gc variable regions, as well as multivalent vaccines for SBV Gc, all of which have shown good immune effects [119]. In addition, OBV vaccines developed based on adenovirus vectors or mRNA vaccines may also be feasible. For example, research on an adenovirus vector vaccine based on RVFV has shown good immune effects. In addition, mRNA vaccines based on the six-mutant RVFV M gene also show good immune effects, which may indicate the prospect and importance of developing Gc-based OBV vaccines [120,121].

This overview details the complex processes involved in OBV cellular infection, emphasizing the glycoprotein Gc, along with dynamin-, clathrin-, and pH-dependent endocytosis pathways and alternative viral entry patterns, culminating in the viral budding and release of OBV particles. Furthermore, investigations into the interplay between Gc and host cellular factors offer additional information which can facilitate our understanding of how OBV regulates host cells to achieve optimal infection. Given the current information gaps, further research on OBV is essential as it will deepen our understanding of its infection mechanisms and provide a solid foundation for developing effective prevention and control strategies.

## Figures and Tables

**Table 1:** Main OBVs that infect humans and animals.

Species	Virus Name	Host	Vector	Clinical Signs	References
*Orthobunyavirus ainoense*	Aino virus (AINOV)	cows	biting midge, mosquito	Hydranencephaly	[19,20]
*Orthobunyavirus akabaneense*	Akabane virus (AKAV)	cows, sheep, goats, pigs	biting midge, mosquito	Abortion and neonatal hydranencephaly	[21,22]
*Orthobunyavirus bataiense*	Batai virus (BATV)	humans, cows, sheep, goats, rodents, birds	mosquito	Meningoencephalitis	[5]
*Orthobunyavirus bunyamweraense*	Bunyamwera virus (BUNV)	humans, horses	mosquito	CNS disease and abortion	[23,24]
*Orthobunyavirus bunyamweraense*	Germiston virus (GERV)	humans	mosquito	Mental confusion	[14]
*Orthobunyavirus cacheense*	Cache Valley virus (CVV)	humans, sheep	mosquito	Encephalitis and meningitis (acute and chronic)	[25,26]
*Orthobunyavirus encephalitidis*	California encephalitis virus (CEV)	humans, horses	mosquito	Encephalitis	[27]
*Orthobunyavirus jamestownense*	Jamestown Canyon virus (JCV)	humans	mosquito, horse fly	Meningitis and meningoencephalitis	[28]
*Orthobunyavirus jamestownense*	Inkoo virus (INKV)	humans	mosquito	Encephalitis and meningitis	[29]
*Orthobunyavirus lacrosseense*	La Crosse virus (LACV)	humans, dogs	mosquito	Encephalitis and meningitis	[30,31]
*Orthobunyavirus oropoucheense*	Oropouche virus (OROV)	humans	biting midge, mosquito	Encephalitis and meningitis	[17,32]
*Orthobunyavirus schmallenbergense*	Schmallenberg virus (SBV)	cows, sheep, goats	biting midge	Abortion and neonatal hydranencephaly	[33,34,35]
*Orthobunyavirus shuniense*	Shuni virus (SHUV)	humans, cows, horses, sheep, goats	biting midge, mosquito	Encephalitis and meningitis	[36,37,38]
*Orthobunyavirus tahynaense*	Ťahyňa virus (TAHV)	humans	mosquito	Encephalitis and meningitis	[39]
*Orthobunyavirus tensawense*	Tensaw virus (TENV)	humans, foxes	mosquito	Encephalitis and rabies-like symptoms	[40,41,42]

## Data Availability

Not applicable.
